# Hemolysis Is Associated with Low Reticulocyte Production Index and Predicts Blood Transfusion in Severe Malarial Anemia

**DOI:** 10.1371/journal.pone.0010038

**Published:** 2010-04-06

**Authors:** Rolf Fendel, Christian Brandts, Annika Rudat, Andrea Kreidenweiss, Claudia Steur, Iris Appelmann, Bettina Ruehe, Paul Schröder, Wolfgang E. Berdel, Peter G. Kremsner, Benjamin Mordmüller

**Affiliations:** 1 Medical Research Unit, Albert Schweitzer Hospital, Lambaréné, Gabon; 2 Institute of Tropical Medicine, University of Tübingen, Tübingen, Germany; 3 Department of Pharmacology, University of Illinois College of Medicine, Chicago, Illinois, United States of America; 4 Department of Medicine, Hematology/Oncology, University of Münster, Münster, Germany; 5 Department of Medicine, Hematology/Oncology, Goethe-University, Frankfurt, Germany; University of California Los Angeles, United States of America

## Abstract

**Background:**

Falciparum Malaria, an infectious disease caused by the apicomplexan parasite *Plasmodium falciparum*, is among the leading causes of death and morbidity attributable to infectious diseases worldwide. In Gabon, Central Africa, one out of four inpatients have severe malarial anemia (SMA), a life-threatening complication if left untreated. Emerging drug resistant parasites might aggravate the situation. This case control study investigates biomarkers of enhanced hemolysis in hospitalized children with either SMA or mild malaria (MM).

**Methods and Findings:**

Ninety-one children were included, thereof 39 SMA patients. Strict inclusion criteria were chosen to exclude other causes of anemia. At diagnosis, erythrophagocytosis (a direct marker for extravascular hemolysis, EVH) was enhanced in SMA compared to MM patients (5.0 arbitrary units (AU) (interquartile range (IR): 2.2–9.6) vs. 2.1 AU (IR: 1.3–3.9), p<0.01). Furthermore, indirect markers for EVH, (i.e. serum neopterin levels, spleen size enlargement and monocyte pigment) were significantly increased in SMA patients. Markers for erythrocyte ageing, such as CD35 (complement receptor 1), CD55 (decay acceleration factor) and phosphatidylserine exposure (annexin-V-binding) were investigated by flow cytometry. In SMA patients, levels of CD35 and CD55 on the red blood cell surface were decreased and erythrocyte removal markers were increased when compared to MM or reconvalescent patients. Additionally, intravascular hemolysis (IVH) was quantified using several indirect markers (LDH, α-HBDH, haptoglobin and hemopexin), which all showed elevated IVH in SMA. The presence of both IVH and EVH predicted the need for blood transfusion during antimalarial treatment (odds ratio 61.5, 95% confidence interval (CI): 8.9–427). Interestingly, this subpopulation is characterized by a significantly lowered reticulocyte production index (RPI, p<0.05).

**Conclusions:**

Our results show the multifactorial pathophysiology of SMA, whereby EVH and IVH play a particularly important role. We propose a model where removal of infected and non-infected erythrocytes of all ages (including reticulocytes) by EVH and IVH is a main mechanism of SMA. Further studies are underway to investigate the mechanism and extent of reticulocyte removal to identify possible interventions to reduce the risk of SMA development.

## Introduction

Malaria due to *Plasmodium falciparum* is the most important parasitic disease worldwide; approximately 270 million people get infected and 1 to 3 million deaths occur every year, most of them in sub-Saharan Africa [1]. Most severely affected are children up to the age of 5 years [2]. Complications leading to death include severe malarial anemia (SMA), cerebral malaria, hypoglycemia, lactic acidosis and convulsions. SMA is particularly common in regions with high malaria endemicity. The World Health Organization defines SMA as hemoglobin (Hb) level of below 5 g/dl plus a parasite count of more than 10,000 per µl blood [3]. In Lambaréné, Gabon, a hyperendemic area, SMA is one of the most common complications of malaria which requires adjunct therapy [4]. An epidemiological survey undertaken at the same time period than the presented study showed that 25% of all 1 month to 10 years old hospitalized malaria patients had severe anemia, 95% of whom were younger than 5 years old [5]. The recent literature shows a decline of malaria incidence both in urban and rural regions of Gabon, which is the same in some other African regions [6], [7], [8]. This decrease of malaria incidence might indicate the success of recent drug policies and of prevention measures like treated bed nets. In The Gambia, the overall decline of malaria endemicity also led to a reduced frequency of SMA [7]. Nevertheless, recent non-published surveys show that the frequency of SMA in Lambaréné stays rather stable. This might be explained by the supraregional importance of the Albert Schweitzer Hospital in Lambaréné. The severe cases often come from small villages located far away from the hospital, which are not covered by national malaria prevention programs.

Treatment of SMA consists of blood transfusion and antimalarial treatment. In most sub-Saharan African countries, safe blood transfusions are difficult to perform because of inadequate blood banks, difficult access to well tested donors, and prejudices due to a bad reputation of blood transfusions [9]. In addition, testing, processing, and storage of blood products is generally far from a high standard and poses a substantial risk of transfusion reactions and transmission of infectious diseases [10]. To avoid the risk associated with blood transfusions it was proposed to postpone transfusion as long as SMA patients are clinically stable [2], [11], [12]. Other measures to reduce the need for transfusions than risk-group identification have not been tested thus far.

A rational approach to identify alternative treatments is difficult because the etiology of SMA is not known. Current hypotheses about the development of SMA were reviewed recently [13], [14], and the only consensus is about the lack of detailed knowledge we have, especially because animal models are only partly informative and few clinical studies investigated SMA *sensu stricto* (Hb concentration <5 g/dl or hematocrit <15% and no other cause of anemia).

Generally, disturbance of the equilibrium between production and clearance of red blood cells (RBCs) leads to anemia. Although decreased erythropoiesis may have an additive role [15], [16], cumulated data suggests that enhanced clearance of RBCs is critical for the development of SMA. Direct parasite-mediated destruction of RBCs does not explain the amount of anemia in SMA and mathematical modeling predicts that lysis of non-parasitized erythrocytes has an important role in the development and the degree of anemia [17], [18], which confirms the clinical notion that SMA in African children is not associated with high parasitemias. Old or altered RBCs are removed by phagocytosis (extravascular hemolysis, EVH) or lysis (intravascular hemolysis, IVH), although in most hemolytic disease states both mechanisms act at the same time. EVH is usually deduced from indirect and imprecise markers such as spleen size, whereas IVH can be estimated from serum concentrations of biomarkers such as haptoglobin, hemopexin, lactate dehydrogenase (LDH), or alpha-hydroxybutyrate dehydrogenase (α-HBDH). We recently developed a method to measure erythrophagocytic activity of monocytes from malaria patients more accurately [19] and used it in the present study to characterize RBC removal during SMA.

Several mechanisms have been proposed to contribute to RBC destruction during malaria, including increased release of oxygen radicals [20], presumably leading to stiffening of the RBC membrane [21], [22], and exhaustion of complement receptor 1 (CD35) and decay accelerating factor (CD55) on RBC membranes, which leads to sensitization for phagocytosis [23], [24]. All these concepts postulate an “accelerated aging” phenotype of RBCs during malaria that leads to clearance of RBCs by unspecific mechanisms. If, in contrast, predominantly reticulocytes and young RBCs would be removed from the circulation (such as in thalassemias) by a malaria-induced factor, its contribution to the development of SMA would be disproportionally high and might explain the strong effect of antimalarial treatment alone on recovery from SMA [25].

In the present report we show that SMA patients have increased levels of erythrophagocytosis and IVH. The odds for a blood transfusion in addition to antimalarial treatment are high when erythrophygocytosis plus IVH are elevated. Interestingly, these patients are characterized by significantly low levels of reticulocytes. This indicates that the age-structure of the removed RBC population is crucial for the development of clinically unstable SMA. Understanding the etiology of this might lead to an alternative management of this complication.

## Materials and Methods

### Ethics statement

This study was conducted according to the principles expressed in the Declaration of Helsinki and approved by the ethics committees of the International Foundation for the Albert Schweitzer Hospital in Lambaréné as well as the ethics committees of the Universities of Münster and Tübingen, Germany. Written informed consent was obtained from parents or guardians of all 91 study patients. All patients were children of pre-school age and, in addition to written informed consent by the parent, assent was sought where possible.

### Patients and study design

**Figure 1:**
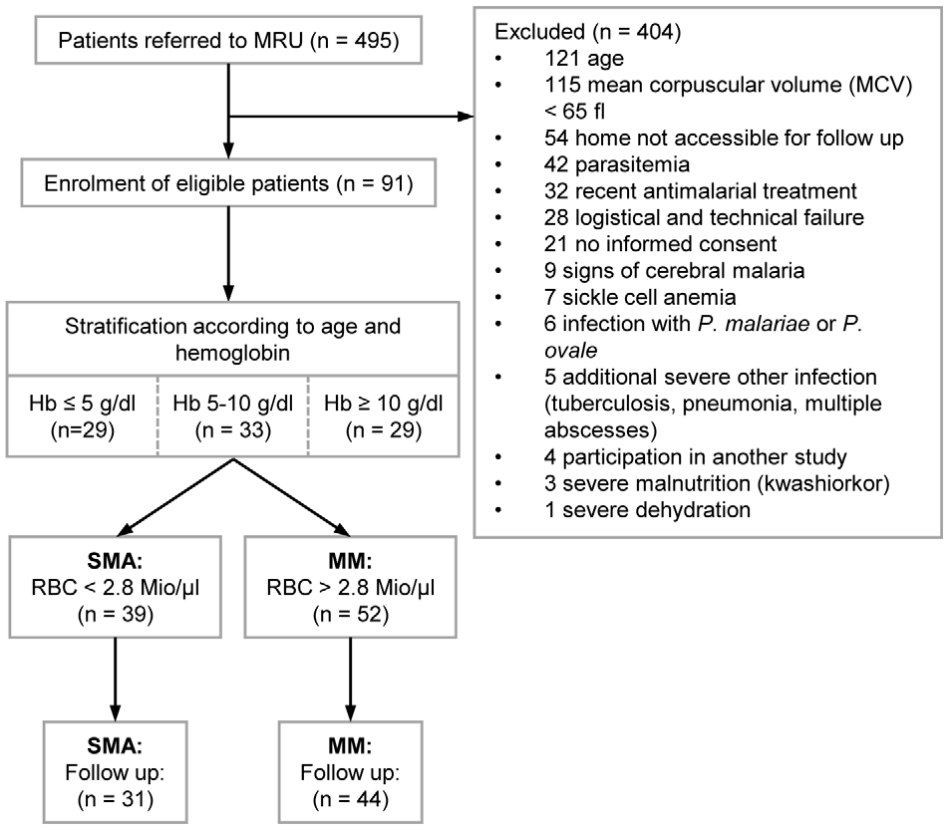
Study-flow chart.

The study took place at the Medical Research Unit (MRU) of the Albert Schweitzer Hospital in Lambaréné, Gabon between December 2003 and July 2005. In total 91 children, aged between one and six years, were admitted to the study. Admission criteria were positive thick blood smears of above 1,000 *P. falciparum* parasites per µl, mean corpuscular volume above 65 fl and no sickle cell disease. Patients were recruited and stratified into three Hb-concentration strata with matched age between strata (Figure 1). For statistical analysis, patients were allocated to a severe malarial anemia group (SMA, RBC <2.8 Mio/µl), and a moderate malarial anemia group (RBC >2.8 Mio/µl). All patients were hospitalized and treated with quinine and clindamycin, the current standard for the treatment of SMA at the Albert Schweitzer Hospital [26]. Patients received blood transfusion or any other supportive treatment upon clinical judgment of the clinician. Patients were actively followed up for two months (treatment period day 0 to day 4, control visits on day 14, day 28 and day 56) to allow hematological values to return to baseline. If they had a parasitemic episode during follow-up, patients were followed for another 2 months after their last infection. From each patient, 5 ml of heparinized blood and 1 ml of EDTA blood was obtained directly before treatment antimalarial treatment and blood transfusion. Total leukocyte counts and RBC values were measured using a Cell Dyn 3000 (Abbott). Differential blood counts and counting of hemozoin - positive phagocytes were done on thin blood smears [27], [28]. Reticulocyte counting was done on brilliant cresyl blue stained samples, and reticulocyte production index was calculated according to standard procedures [29]. The presence of hemoglobin F, S or C was assessed by hemoglobin electrophoresis and Kato-Katz smears were done to test for intestinal parasites including hookworm infections. Spleen volume was measured by ultrasonography on admission and after reconvalescence. The relative spleen volume was calculated as the quotient of spleen volume on admission and after reconvalescence. In addition, palpable subcoastal spleen enlargement was estimated on admission. The simplified Multiple Organ Dysfunction Score (sMODS; Table S1, Table S2, Table S3 and Table S4) was estimated as previously described [30]. Care - givers of the patients were asked for the duration of illness of the children and detailed clinical history was taken.

### Phagocytosis assay

Blood was processed within one hour after sampling and phagocytosis assay were done as previously described [19]. Briefly, monocytes were isolated by CD14^+^ positive selection using magnetic cell sorting (Miltenyi). RBCs from the same patient and, for the positive and negative control, from one malaria naïve 0+ voluntary blood donor were centrifuged, washed and stained with 5 nM carboxy-fluorescein-diacetate-succinimidyl ester (CFDA). One batch of control RBCs was opsonized with human anti-D IgG (1∶20) to serve as a positive control, the other was left untreated to serve as negative control. The patients' RBCs were not further treated. For phagocytosis assay, 2×10^5^ monocytes were incubated for 4 hours at 37°C/5% CO_2_ with 100 fold numeric excess of the respective RBC preparation in RPMI plus 10% fetal calf serum. After incubation, non-phagocytozed RBCs were lysed and monocytes containing ingested RBCs were analyzed by flow cytometry. Data was expressed as relative phagocytosis to negative control. Hence, data is presented as relative phagocytosis activity for positive control and the sample from the same patient (autologous RBCs).

### Surface staining of RBCs

Surface markers of RBCs were measured by flow cytometry. All antibodies were obtained from BD Biosciences if not stated otherwise. Fifty µl blood were washed once in phosphate buffered saline complemented with 2 mM EDTA and 0.5% BSA (PBS-EB) and 5 µl of RBC pellet was stained subsequently with one of the following antibodies: FITC anti-human C3c (DakoCytomation), FITC anti-human CD35, FITC anti human CD55, FITC anti human CD59, FITC anti-human IgG (Biozol), or appropriate isotype controls. Samples were incubated for 30 minutes at room temperature in the dark, washed thrice in PBS and measured by flow cytometry. Another 5 µl of blood was washed once in annexin V binding buffer (10 mM HEPES, pH 7.4, 140 mM NaCl, 5 mM CaCl_2_), stained with annexin V and measured immediately by cytometry.

### Serum parameters

Neopterin (MP Biomedicals) and hemopexin (GenWay Biotech) quantification was performed by ELISA using commercially available kits according to the respective manufacturers' specifications. LDH, α-HBDH and haptoglobin were measured by the central laboratory of the University Hospital Münster according to standard procedures.

### Flow cytometry

Flow cytometry analysis of RBC surface markers and phagocytosis was done on a Partec CyFlow SL Cytometer equipped with a 488 nm laser and detection at 515–550 nm. Data was acquired and analyzed with FlowMax FCM v2.4f (Partec, Germany). Mean fluorescence intensities were quantified using Weasel v2.6 (Cytometry Lab, The Walter and Eliza Hall Institute of Medical Research, Australia). Monocytes and RBCs were gated on FSC – SSC plots, the rate of fluorescent positive cells and fluorescence intensity was acquired on FL-1-histograms.

### Statistical analysis

The study was designed as a case-control study to identify variables that discriminate malaria patients with and without SMA. Variables associated with RBC production, RBC removal, clinical and parasitological presentation were measured and recorded. All patients received the same antimalarial treatment. Recruitment in three age-matched Hb-strata resulted in two populations with different degree of anemia. Post-hoc grouping of severe and non-severe malaria was done by least square fit. Severely anemic patients that required blood transfusions were analyzed as a subgroup. All statistical analysis was done using JMP v5.0.1 (SAS Institute) and R v2.7 [31]. Level of significance was set at two-sided p = 0.05 for all tests. Kruskal Wallis rank sum test was used to compare continuous data among groups. Adjustment for confounders (weight, age) has been carried out by logistic regression analysis where indicated. Values indicated are medians and interquartile ranges, if not stated otherwise. Counting data was analyzed by χ^2^ or Fisher's exact test.

## Results

### Patients

Throughout the study 495 malaria patients were referred to the MRU and 91 patients were admitted to this study. Main causes for exclusion were non-matching age, low MCV (an indicator of iron deficiency or thalassemia), and logistics. At inclusion, eligible patients were stratified according to age and hemoglobin concentration (Figure 1). Analysis of the distribution of RBC numbers in the study population showed a bimodal pattern. Analysis of the study population was done on a grouped dataset using a least square fit of RBC number on two normal distributions to discriminate anemic (SMA; severe malarial anemia) *versus* non-anemic (MM; mild malaria) children with a cut-off value of 2.8×10^6^ RBCs/µl.

On admission no significant difference in parasitemia, age, height, and MCV between SMA and MM patients was observed. Those with SMA weighted less than MM patients, an observation that is most probably due to dehydration as on follow-up no significant difference was found (p = 0.06). To exclude chronic malnutrition as contributor to SMA, z-scores for “height for age”, “weight for height”, and “weight for age” were estimated. No significant differences were found in any of these scores (data not shown). As expected, only SMA patients received blood transfusions (n = 17) and the percentage of reticulocytes was significantly higher in the SMA compared to the MM group (Table 1). Sickle-cell trait carriers were equally distributed between the groups. Two SMA patients died. A 3 year old boy presented with respiratory distress and signs of acute renal insufficiency and died on the first day after admission. The second, a 22 month old boy, died in a rural hospital about 200 km from Lambaréné two months after discharge from the MRU. Extensive efforts to discover the cause of death by verbal autopsy and studying the patient's records did not reveal an obvious explanation.

**Table 1:** Clinical data of anemic and non-anemic patients on admission.

	Total study population (n = 91)	Group MM (n = 52)	Group SMA (n = 39)	p-value
**Hb (g/dl)**	8.0 (4.9–10.0)	10 (8.7–10.5)	4.9 (4.4–5.3)	-a
**Hct (%)**	23.9 (14.9–29.0)	28.1 (25.9–30.6)	14.3 (12.3–16.0)	-a
**RBCs (10^6^ cells/µl)**	3.27 (2.19–3.90)	3.87 (3.50–4.30)	1.93 (1.60–2.30)	-a
**Parasitemia (per µl)**	20,200 (6,960–76,000)	23,800 (8,880–69,000)	12,000 (5,880–101,600)	0.81^b^
**Reticulocytes (%)**	2.4 (1.4–5.2)	1.8 (1.3–2.5)	5.3 (2.3–10.6)	<0.0001^b^
**MCV (fl)**	72.6 (68.6–76.8)	71.8 (67.9–76.0)	74.0 (69.3–78.1)	0.15^b^
**Age (months)**	32 (22–50)	36 (23–53)	27 (20–43)	0.13^b^
**Weight (kg)**	13.0 (10.8–15.0)	13.5 (11.0–15.0)	11.4 (9.8–14.0)	0.04^b^
**Height (cm)**	89 (81–100)	90 (83–104)	87 (76–96)	0.12^b^
**# Sex (male/female)**	45/46	26/26	19/20	1^c^
**# Sickle cell trait**	8	6	2	0.46^c^
**# Blood transfusion**	17	0	17	<0.0001^c^

MM: mild malaria, SMA: severe malarial anemia, Hb: hemoglobin, Hct: hematocrit, RBCs: red blood cells, MCV: mean corpuscular volume, results are in median (interquartile ranges) and count data (#).

a parameter used for group definition - no p-value calculated, ^b^Kruskal-Wallis rank sum test, ^c^χ^2^-test.

### Red blood cell turnover

#### Extravascular hemolysis

**Figure 2:**
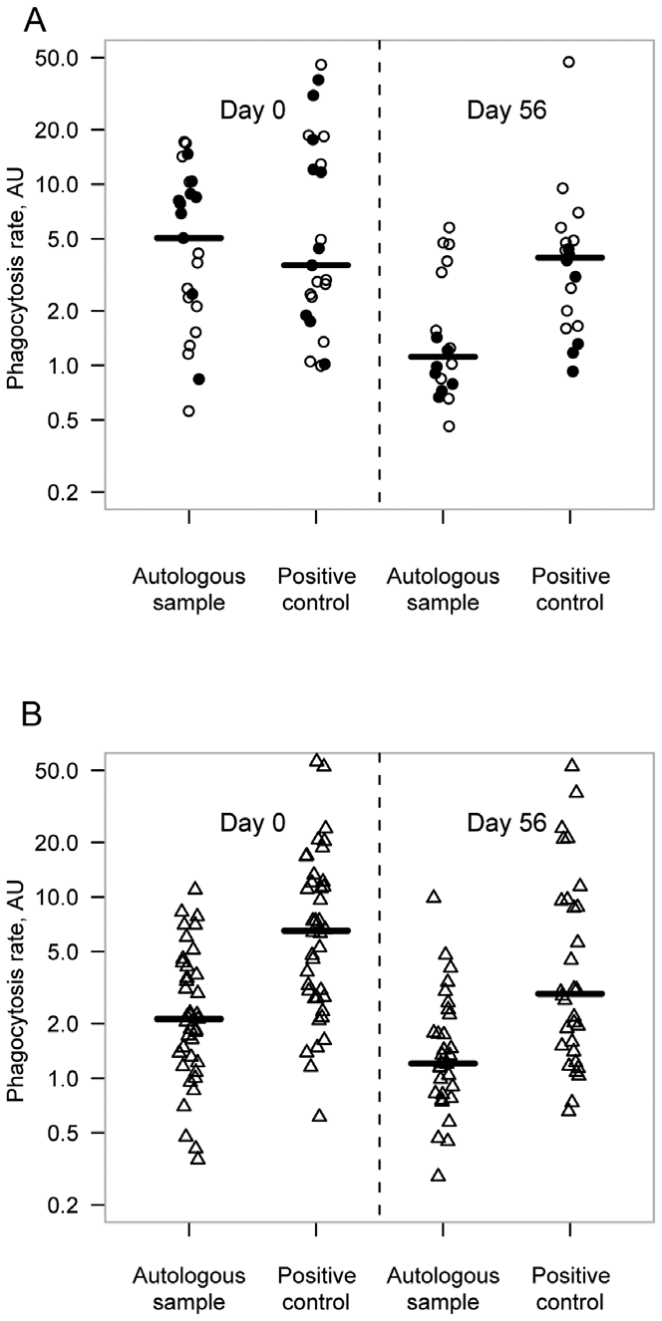
Phagocytosis rate in severe malarial anemia (SMA) and mild malaria (MM) patients: The plot shows individual values of relative phagocytosis of autologous samples (patients monocytes phagocytosing autologous RBCs) and positive control samples (patients monocytes phagocytosing anti-D IgG opsonized control RBCs). Thick lines represent median values. Phagocytosis rates were investigated on admission and after reconvalescence on day 56. In A) phagocytosis rates are shown for SMA patients. Phagocytosis rates of autologous samples at day 0 are as high as positive control. On day 56, autologous sample is significantly lower than positive control (p<0.05, ○ SMA - non-transfused, • SMA - transfused). In B), phagocytosis rates of MM patients are illustrated. Phagocytosis rates of autologous samples are significantly lower on day 0 as well as on day 56 (p<0.05, Δ MM).

To estimate the level of erythrophagocytosis, monocytes were isolated and incubated with fluorescently labeled RBCs from the patient (“autologous RBCs”) or from a healthy donor with (“positive control”) or without (“negative control”) opsonisation with human anti-D IgG antibodies. On admission, erythrophagocytosis of autologous RBCs was higher in SMA (median: 5.0 AU) compared to MM patients (median: 2.1 AU, p<0.01), whereas in reconvalescent children, erythrophagocytosis was not significantly different (p = 0.94; Figure 2a and b). Erythrophagocytosis of positive control RBCs was similar in all conditions tested and within the range of autologous RBCs from SMA patients on admission (median: 4.3 AU).

Spleen size, a rough estimate of *in vivo* phagocytic activity, was increased in SMA patients on admission. Other markers of monocyte activation such as neopterin [32] (p<0.05) and the number of hemozoin containing monocytes on thin blood smears (p<0.0001) were also significantly different between SMA and MM children (Table 2).

**Table 2:** Surrogate markers for extravascular hemolysis and disease severity on admission.

	Total study population (n = 91)	Group MM (n = 52)	Group SMA (n = 39)	p-value^a^
**Pigment in Monocytes (%)**	10 (4–20)	6 (2–10)	19 (9–28)	<0.0001
**Relative spleen volume**	2.7 (2.0–3.8)	2.5 (1.9–3.1)	3.0 (2.5–4.2)	<0.05
**Spleen (cm)**	2 (0–3)	1 (0–2)	2 (2–4)	<0.0001
**Neopterin (ng/ml)**	7.2 (5.7–9.9)	6.5 (5.3–8.6)	8.1 (6.4–12.3)	<0.05
**Lactate (mmol/l)**	2.6 (2.0–3.3)	2.3 (1.8–2.9)	2.9 (2.3–4.3)	<0.005
**sMODS**	14 (13–15)	13 (12–14)	15 (14–17)	<0.0001

MM: mild malaria, SMA: severe malarial anemia, relative spleen volume: calculated as spleen volume at recruitment/spleen volume after reconvalescence (measured by sonography), Spleen (cm) represents subcoastal spleen enlargement, sMODS: simplified multi organ dysfunction score, results are in median (interquartile ranges).

a Kruskal-Wallis rank sum test.

#### Removal tags on RBCs

A possible cause of enhanced erythrophagocytosis is the exposure of removal signs on the RBC surface (Table 3). CD35 and CD55, two complement receptors on the surface of RBCs were significantly down-regulated in SMA patients, whereas annexin V binding was increased in anemic patients. Expression of CD59 and C3 - fragment binding on the surface were not significantly different between the two groups. No association of CD35 or CD55 expression with age was observed.

**Table 3:** Erythrocyte surface makers on admission.

	Total study population (n = 91)	Group MM (n = 52)	Group SMA (n = 39)	p-value^a^
**Annexin V (%)**	0.6 (0.3–1.3)	0.5 (0.3–0.7)	1.1 (0.6–1.9)	<0.005
**CD35 (MFI)**	4.1 (3.9–4.8)	4.3 (4.0–5.0)	4.0 (3.7–4.5)	<0.05
**CD55 (MFI)**	5.8 (5.1–7.1)	6.4 (5.5–7.8)	5.5 (4.8–6.5)	<0.05
**CD59 (MFI)**	53.3 (44.5–71.0)	51.4 (44.5–68.5)	56.3 (44.1–71.0)	0.82
**C3c (MFI)**	3.5 (1.2–5.8)	3.6 (3.2–4.3)	3.6 (3.5–4.0)	0.55
**IgG (%)**	4.6 (4.0–5.6)	4.3 (3.9–5.3)	5.0 (4.0–5.9)	0.08

MM: mild malaria, SMA: severe malarial anemia, %: percentage of fluorescence positive cells, MFI: mean fluorescence intensity, IgG: immunoglobulin G, results are in median (interquartile ranges).

a Kruskal-Wallis rank sum test.

#### Intravascular hemolysis on admission

In addition to EVH, IVH might contribute to the development of SMA. To investigate IVH we measured haptoglobin, hemopexin, LDH, α-HBDH, and hemoglobinuria. LDH and α-HBDH concentrations were increased in SMA compared to MM patients whereas haptoglobin and hemopexin concentrations were lower (Table 4). No significant difference between the groups was present with respect to hemoglobinuria (p = 0.28).

**Table 4:** Markers of intravascular hemolysis on admission.

	Total study population (n = 91)	Group MM (n = 52)	Group SMA (n = 39)	p-value^a^
**Haptoglobin (mg/dl)**	0 (0–73.5)	41 (0–110)	0 (0–0)	<0.0001
**Hemopexin (mg/dl)**	8.7 (4.8–10.8)	10.0 (8.8–11.3)	4.2 (1.9–6.3)	<0.0001
**LDH (U/l)**	432 (325–582)	350 (309–408)	579 (454–858)	<0.0001
**α-HBDH (U/l)**	355(281–523)	298 (250–348)	522 (414–694)	<0.0001

MM: mild malaria, SMA: severe malarial anemia, LDH: lactate dehydrogenase, α-HBDH: alpha hydroxybutyrate dehydrogenase, results are in median (interquartile ranges).

a Kruskal-Wallis rank sum test.

#### Interaction of IVH and erythrophagocytosis

**Figure 3:**
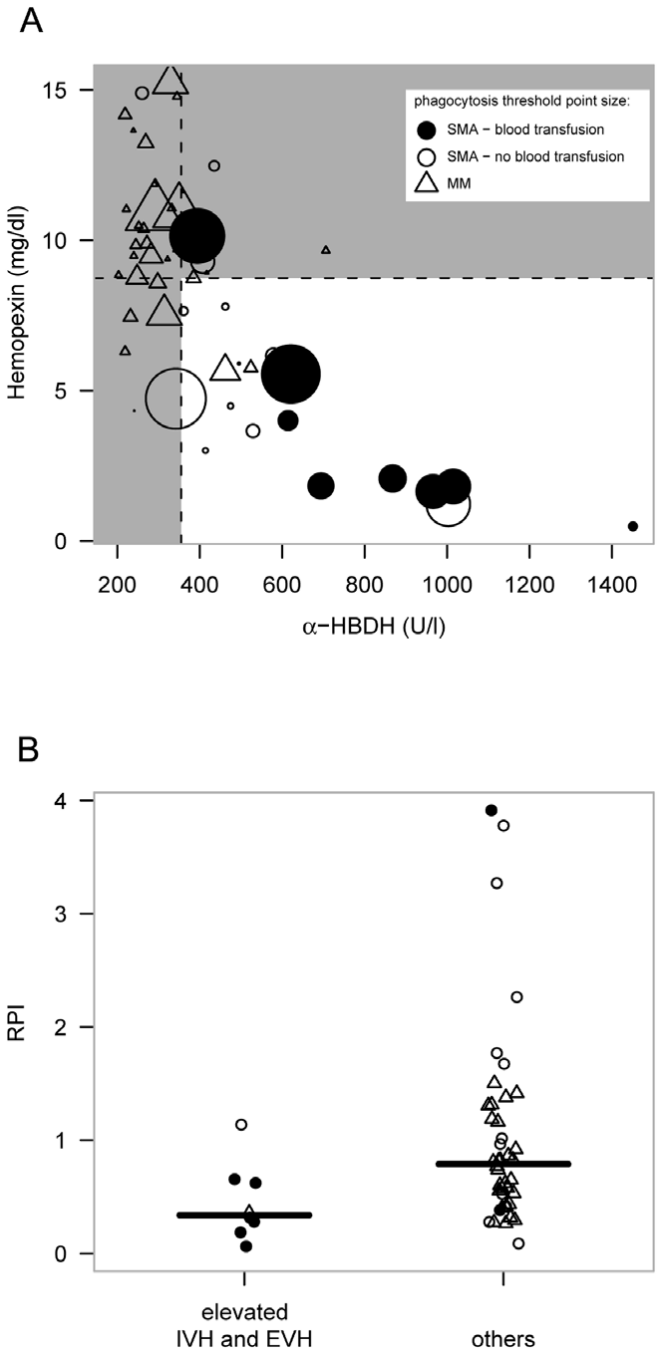
Distribution of enhanced intravascular and extravascular hemolysis across the patient groups and its correlation with reduced RPI: In figure A), a dot plot of α-HBDH vs. hemopexin concentration, both makers for IVH, at presentation is shown on the x and y axis, respectively. The size of the data symbols represents erythrophagocytosis activity, a direct marker of EVH (the bigger the symbol, the higher the autologous erythrophagocytosis). Classification criteria for “elevated IVH and EVH” match for those symbols bigger than the borderline levels shown in the plot and laying in the white region. Figure B: The Group having both “elevated EVH and IVH” had significantly lower reticulocyte productions indices than the other patients (p<0.05). Δ MM; ○ SMA - non transfused; • SMA - blood transfused.

IVH - EVH interaction and its effect on the severity of anemia was investigated. To do this, thresholds for elevated intravascular and extravascular hemolysis were defined and patients were grouped accordingly. IVH was estimated by two variables: hemopexin and α-HBDH. High IVH was defined as a hemopexin level below the median of the all patients (8.7 mg/dl) plus an α-HBDH of above median level (355 U/l). A combination of both factors increases specificity of the IVH estimate. Autologous erythrophagocytosis activity represented EVH. The threshold for elevated phagocytosis was defined by the median positive control erythrophagocytosis level seen in all patients (4.3 AU). Figure 3a shows the interaction between those three variables. Patients with enhanced hemolysis in all three variables were grouped together (high hemolysis; HH) and compared to the remaining individuals.

HH patients had a median of 1.46×10^6^ RBCs/µl (IR: 1.28×10^6^–2.14×10^6^ RBCs/µl), while patients with low hemolysis had a median of 3.58×10^6^ RBCs/µl (IR: 2.54×10^6^–4.23×10^6^ RBCs/µl, p<0.001). Most notably, HH patients had an elevated risk to receive a blood transfusion during hospitalization (odds ratio 61.5 (CI: 8.9–427)). Additionally, HH patients had a significantly elevated sMODS of 17 [IR: 16–19] *versus* 12 [IR: 11–13] (p<0.0005) in non-HH patients. High hemolysis was not associated with any of the erythrocyte age markers investigated (data not shown). Hence erythrocyte aging cannot explain the higher odds for blood transfusion in these patients.

The reticulocyte production index (RPI) is a marker for erythropoietic output. It estimates the quantity of reticulocytes in the peripheral blood, corrected for the degree of anemia and therefore is a good measure of whether the reticulocyte production is adequate for the degree of anemia present [29]. In our patient population, HH - children had a significantly reduced RPI (figure 3b, p<0.05, logistic regression). This finding indicates that a subpopulation of malaria patients with high IVH plus EVH have a high risk of receiving blood transfusions and suffer from inadequately low reticulocyte numbers.

### Follow-up

Markers of disease were measured again after 2 months of a parasite-free period. All measured variables were not significantly different between SMA and MM (data not shown). The two groups evolved into one homogeneous population with little variability. Hemoglobin levels raised to 10.6 g/dl, the reticulocyte counts dropped back to normal levels. In both, the SMA and MM group, phagocytosis levels were significantly lower at day 56 than at day 0.

## Discussion

The etiology of severe malarial anemia, one of the most frequent complications in malaria, recently regained interest. Several mechanisms seem to contribute to anemia during malaria, with pronounced differences in different patient populations and animal models. Proposed key mechanisms are suppressed erythropoiesis [15], removal of parasitized erythrocytes [33], [34], enhanced clearance of both parasitized and unparasitized erythrocytes by the spleen and other phagocytes [35], [36], [37], [38], and intravascular lysis [23]. The relative contribution and interaction of these individual factors on SMA is unknown. Recently, an interesting and challenging hypothesis was presented on the basis of studies in humans [35], [37], [39]: complement and complement regulatory proteins are dysregulated in SMA and lead to enhanced removal of erythrocytes because complement-binding receptors (CD35 and CD55) on erythrocytes have been consumed by excessive amounts of activated complement in serum and leave the RBC prone to lysis. In addition, this finding provides an explanation for the increased risk to develop SMA in infancy: children between 1 and 3 years constitutively express reduced levels of complement regulators on their RBC surface and therefore the probability to develop SMA is higher [40]. Nevertheless, these results were not confirmed in a subsequent study from Ghana [23], where no correlation of CD35 and CD55 expression levels and anemia status were found. Other proposed mechanisms are based on reduced deformability of RBCs as an effect of oxidative damage due to proinflammatory cytokines and parasite products [22], [41].

In this study we reproduced some findings of Waitumbi *et al*. [35], [37], [39]: SMA patients had reduced CD35 and CD55 expression on the RBC surface. In addition, we observed enhanced *ex vivo* erythrophagocytosis in SMA patients. This confirms results from previous pilot studies [19]. Increased *ex vivo* erythrophagocytosis is accompanied by surrogate markers of phagocyte activation like neopterin and spleen size. However, we were unable to detect an association between complement or immunoglobulin deposition on freshly isolated RBCs. Furthermore, CD35 and CD55 RBC surface expression was not associated with age. In contrast to complement- and immunoglobulin-binding, SMA children showed elevated phosphatidylserine exposure on RBC surface, a removal marker that tags old or damaged RBC for phagocytosis [42], [43]. In SMA patients the rate of RBC removal outcompetes the rate of RBC production. Based on a static model it has been proposed that half of the drop of hematocrit due to malaria occurred before admission [44]. This direct interpretation may be oversimplistic because dynamic rates (and not a fixed absolute number of removed erythrocytes) determines the development of anemia. Turn-over of erythrocytes follows complex (possibly non-linear) dynamics [45] and future research may provide us with new insights.

Most hypotheses on the development of SMA and removal of infected RBC rely on the assumption that mature and old RBCs are removed from the circulation by EVH and IVH. Our results point to an alternative direction: association of high hemolysis (HH) with low RPI infers an important effect on reticulocytes and young RBCs in SMA. Although we cannot exclude that in addition erythropoiesis is negatively affected in SMA patients, this finding is of particular importance because removal of reticulocytes and young erythrocytes (which contribute disproportionately to the total number of circulating erythrocytes in SMA patients) by erythrophagocytosis will stunt compensatory erythropoiesis.

We can only speculate on the mechanism of reticulocyte removal. Possibly, stress erythropoiesis, a phenotype present in diseases like thalassemia, may be involved. In thalassemia, erythropoietic cells are oxidatively stressed by the accumulation of precipitated α-globin-chain tetramers. Consequently, only few erythropoietic cells undergo full maturation and are removed as reticulocytes either in the bone marrow or the spleen. Hemozoin, a parasite byproduct released at the burst of infected erythrocytes after schizogeny, has been proposed to contribute to SMA. It has been proposed that hemozoin may, together with an elevated TNF-α concentration, inhibit erythropoiesis [15], [46]. An effect of hemozoin on early erythropoietic progenitors was not the focus of this study, but an effect on reticulocytes and young circulating RBC might be present through the hemozoin-mediated oxidative stress on RBC membranes [47]. Since SMA in African children is frequently a chronic disease, very low RBC numbers are supported. This observation led to the clinical practice to delay blood transfusions as long as possible unless patients are clinically unstable. In line with our hypothesis of preferential (or at least equivalent) removal of young erythrocytes and therefore stunting of compensatory erythropoiesis in SMA, we observed that high IVH and high EVH increased odds for blood transfusion and associates with low RPI. So far, increased IVH and EVH in SMA were attributed to the lysis of infected and prematurely aged RBCs. Surprisingly little is known about the age structure of RBC populations in SMA.

A drawback of our study design is that we cannot explore the early phases of disease development, since SMA is not amenable to a prospective study design because the required close follow up to detect early hematological changes prevents the development of SMA [40].

This study investigated young children in a hyperendemic area in Central Africa, one of the populations mostly affected by SMA. Stringent selection of patients and control or exclusion of interfering factors such as age, iron deficiency, malnutrition or hemoglobinopathies allowed the specific investigation of SMA. In conclusion, we propose that hemolytic removal of reticulocytes and young RBCs stunts compensatory erythropoiesis and therefore is a critical step for the development of clinically unstable SMA. Quantitative studies of the age pattern of removed erythrocyte populations and discovery of molecular events that determine SMA-associated removal of reticulocytes should give new and interesting insight in the development of SMA and might lead to urgently needed new interventions.
